# Ketogenic Diet High in Saturated Fat Promotes Colonic Claudin Expression without Changes in Intestinal Permeability to Iohexol in Healthy Mice

**DOI:** 10.3390/nu16010018

**Published:** 2023-12-20

**Authors:** Lotta Toivio, Hanna Launonen, Jere Lindén, Markku Lehto, Heikki Vapaatalo, Hanne Salmenkari, Riitta Korpela

**Affiliations:** 1 Department of Pharmacology, Faculty of Medicine, University of Helsinki, 00014 Helsinki, Finland; hanna.launonen@helsinki.fi (H.L.); heikki.vapaatalo@helsinki.fi (H.V.); 2 Human Microbiome Research Program, Faculty of Medicine, University of Helsinki, 00014 Helsinki, Finland; 3 Department of Veterinary Biosciences, Faculty of Veterinary Medicine, University of Helsinki, 00014 Helsinki, Finland; jere.linden@helsinki.fi; 4 Finnish Centre for Laboratory Animal Pathology, Helsinki Institute of Life Science, University of Helsinki, 00014 Helsinki, Finland; 5 Folkhälsan Institute of Genetics, Folkhälsan Research Center, 00290 Helsinki, Finland; markku.lehto@helsinki.fi (M.L.); hanne.salmenkari@helsinki.fi (H.S.); 6 Department of Nephrology, University of Helsinki and Helsinki University Hospital, 00290 Helsinki, Finland; 7 Research Program for Clinical and Molecular Metabolism, Faculty of Medicine, University of Helsinki, 00014 Helsinki, Finland

**Keywords:** intestinal permeability, ketogenic diet, dietary fat, tight junction proteins

## Abstract

Ketogenic diets (KDs) have been studied in preclinical models of intestinal diseases. However, little is known of how the fat source of these diets influences the intestinal barrier. Herein, we studied the impact of four-week feeding with KD high either in saturated fatty acids (SFA-KD) or polyunsaturated linoleic acid (LA-KD) on paracellular permeability of the intestine to iohexol in healthy male C57BL/6J mice. We investigated jejunal and colonic tight junction protein expression, histological changes, and inflammatory markers (*Il1b*, *Il6*, *Tnf*, and *Lcn2*), as well as the activity and expression of intestinal alkaline phosphatase (IAP) in feces and jejunal tissue, respectively, and plasma lipopolysaccharide. KDs did not change intestinal permeability to iohexol after two or twenty-six days of feeding regardless of fat quality. SFA-KD, but not LA-KD, upregulated the colonic expression of tight junction proteins claudin-1 and -4, as well as the activity of IAP. Both KDs resulted in increased epithelial vacuolation in jejunum, and this was pronounced in SFA-KD. Jejunal *Il1β* expression was lower and colonic *Il6* expression higher in LA-KD compared to SFA-KD. In colon, *Tnf* mRNA was increased in LA-KD when compared to controls. Overall, the results suggest that KDs do not influence intestinal permeability to iohexol but elicit changes in colonic tight junction proteins and inflammatory markers in both jejunum and colon. Future research will show whether these changes become of importance upon proinflammatory insults.

## 1. Introduction

Proper function of the intestinal barrier is crucial for the absorption of dietary nutrients and the prevention of harmful compounds entering the systemic circulation. While most nutrients are taken up transcellularly, other substances, such as microbial and dietary antigens, mainly pass the epithelial layer through the paracellular pathway only when the integrity of the tight junctions (TJs) connecting adjacent epithelial cells is compromised. This unwanted passage can lead to a vicious cycle where inflammatory cytokines, like tumor necrosis factor α, interleukin 1β, and interleukin 6 are produced, and they further exacerbate barrier dysfunction [1].

Dietary factors can influence the integrity of the epithelial layer and, therefore, the paracellular passage. Some amino acids such as glutamine can decrease intestinal permeability [2,3,4] while gliadin, a component of gluten, may induce dysfunction of the epithelial barrier [5]. Some studies have shown a regular high-fat diet (HFD), which also contains a substantial amount of carbohydrates, to negatively alter the function of the intestinal epithelium [6], and a carbohydrate-free diet with 72% fat to increase permeability [7], although this seems to be dependent on the composition of the microbiota [8]. Regardless, these findings suggest that the amount of dietary fat might be an important nutritional factor in the pathogenesis of barrier dysfunction.

The ketogenic diet (KD) is a dietary approach fundamentally different from regular HFDs in its metabolic effects. While in KD most of the energy is obtained from fat, the amount of carbohydrates is limited (less than 10% of energy). This diet has been used to treat drug-resistant epilepsy for a century [9], in addition to which it is studied for conditions ranging from neurodegenerative diseases [10] to diabetes [11] with encouraging results. The diet might also improve intestinal health through several mechanisms, such as by increasing the circulating levels of the anti-inflammatory ketone body β-hydroxybutyrate (BHB) [12], which is readily used as an energy source by intestinal cells [13], and modulating the gut microbiota [14,15]. Interestingly, the therapeutic effect of KD on epilepsy might also be mediated by its effects on intestinal microbiota [16].

Despite this, preclinical studies on KD and intestinal inflammation have produced inconsistent results. KD promoted the expression of intestinal TJ proteins in a rat model of irritable bowel syndrome [17]. In mice, it protected from experimental colorectal cancer [18] and alleviated dextran sodium sulphate (DSS)-induced colitis [19]. Contrary to this, KD worsened intestinal inflammation in another study with the same model of experimental colitis [20]. The evidence on the impact of different fat sources on intestinal permeability is also mixed. There are studies suggesting that large quantities of the omega-6 fatty acid, linoleic acid (LA), might prime the intestinal barrier for damage upon assault, and that saturated fatty acids (SFAs), especially medium-chain triglycerides, could be less harmful [21,22], while others have reported no or mixed effects [23,24]. It has been theorized that high dietary LA but not SFA may be harmful to the intestine due to increased lipid peroxidation [22]. On the other hand, SFAs have been shown to promote postprandial endotoxemia via increased lipopolysaccharide (LPS) translocation from the gut lumen to the systemic circulation in both animal models [25] and humans [26], which is considered deleterious. While the evidence is inconclusive, it seems that different fat sources have dissimilar, context-dependent effects on intestinal permeability and inflammation.

Our aim was to study whether KDs with different fat sources would have an impact on paracellular permeability and inflammation of the intestine. We fed healthy mice for four weeks with KD comprised of either milkfat high in SFAs, or vegetable fat sources abundant in LA, or with a low-fat control diet. Intestinal permeability to iohexol was determined in vivo at two time points to assess possible differences between two days and four weeks of ketosis and impacts of the diets.

## 2. Materials and Methods

### 2.1. Animal Experiment

The study was approved by the animal research board of the Regional State Administrative Agency for Southern Finland (ESAVI/9377/2019). Seven-week-old male C57BL/6J mice (*n* = 28) were purchased from Scanbur (Karlslunde, Denmark). The study began after 17 days of acclimatization when the animals were nine weeks old. They were kept under a 12 h light–dark cycle at 20 ± 2 °C and 50–60% humidity with *ad libitum* access to food and water.

The mice were housed individually and randomly allocated to three groups based on the dietary intervention: control group (CD) (*n* = 10), high-SFA ketogenic diet group (SFA-KD) (*n* = 9), and high-LA ketogenic diet group (LA-KD) (*n* = 9, of which one had to be euthanized during the study due to progressive weight loss). The diets were custom-made (Envigo, Indianapolis, IN, USA) and matched for protein and micronutrients. The macronutrient compositions of the diets are presented in Table 1.

**Figure 1:**
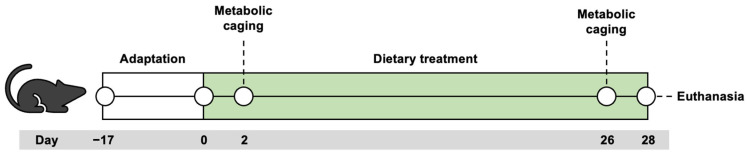
The set-up of the animal experiment.

Food and water consumption was monitored daily. For the first week, the mice were weighed every day, after which their weight was checked every other day. After 28 days of feeding, the mice were euthanized under isoflurane (4%, Vetflurane, Virbac, Carros, France) anesthesia. The set-up of the study is illustrated in Figure 1**.** The duration of the intervention was chosen based on previous experiments showing deleterious effects of high-fat feeding for intestinal barrier after four weeks [7]. 

### 2.2. Measurement of Intestinal Permeability

Paracellular permeability of the intestine was assessed in vivo with iohexol (10 mL/kg, Omnipaque 300^®^, GE Healthcare, Oslo, Norway) after two and twenty-six days of feeding. The mice were weighed, and the solution was administered via a gastric gavage. After this, the animals were placed individually in metabolic cages for 24 h for urine collection. The amount of collected urine was measured, and samples were stored in −80 °C. If fecal contamination was observed, the sample was discarded.

The concentration of iohexol in the urine was measured by enzyme-linked immunosorbent assay (ELISA) according to the instructions of the manufacturer (BioPAL Inc., Worcester, MA, USA). The amount of recovered iohexol was determined as a percentage of the administered amount according to the following equation:

$$\mathrm{Iohexol}\;\left(\%\right)=\frac{\;\mathrm{amount}\;\mathrm{of}\;\mathrm{iohexol}\;\mathrm{recovered}\;\mathrm{in}\;\mathrm{urine}\;\mathrm{in}\;24\;h\;\left(\mathrm{mg}\right)}{\mathrm{amount}\;\mathrm{of}\;\mathrm{administered}\;\mathrm{iohexol}\;\left(\mathrm{mg}\right)}\times 100$$

### 2.3. Collection of Samples

At study termination on day 28, anesthetized animals were sacrificed by drawing blood from *vena cava* into EDTA-tubes (Kisker, Steinfurt, Germany), resulting in the death of the animal. To separate the plasma, samples were centrifuged at 2000× *g* for 15 min at 4 °C, frozen in liquid nitrogen, and stored at −80 °C.

After the euthanasia the entire intestine was removed. 1 cm-long samples were collected from the middle section of jejunum and the proximal and middle section of colon for histological and biochemical analyses. Other samples were opened longitudinally, but one sample from each part of the intestine was left unopened for histological analyses. Colonic pellets were harvested, and the remaining intestinal contents were flushed off with ice cold 0.9% NaCl solution. Except for samples for histological analyses, tissues were snap-frozen in liquid nitrogen and stored at −80 °C.

### 2.4. Histological Analyses

Tissue pieces from jejunum (opened and unopened) and colon (unopened) were fixed in 4% buffered paraformaldehyde (Thermo Fisher Scientific, Waltham, MA, USA) solution for 36 h and then stored at 4 °C in 70% ethanol. The opened jejunal samples were cut into longitudinal halves, and the unopened jejunal parts and colon samples trimmed transversally. The fixed samples were dehydrated, embedded in paraffin, and cut into 4 μm thick sections. All sections were stained with hematoxylin and eosin (HE) stain, and selected jejunal sections with Alcian blue–Periodic Acid–Schiff (AB-PAS) stain, which detects intestinal mucins (goblet cells) and polysaccharides.

A veterinary pathologist (J.L.) read and evaluated the HE-stained slides blinded to the treatments. Vacuolation of the villus epithelium in jejunum was semi-quantitively graded employing three tiers: 0 = no epithelial vacuolation (EV); 1 = mild EV, present only in the basal half of the villi and partly occupying the epithelial cell cytoplasm; 2 = moderate EV, either present only in the basal half of the villi or partly occupying the epithelial cell cytoplasm; 3 = marked EV, present throughout the villi and fully occupying the epithelial cell cytoplasm. Villus *lamina propria* edema was graded as absent (0) or present (1).

### 2.5. Biochemical Assays

The state of ketosis was confirmed by measuring plasma BHB levels with a commercial enzymatic kit (Cayman Chemicals, Ann Arbor, MI, USA). A Limulus Amebocyte Lysate Assay (Pierce^TM^ Chromogenic Endotoxin Quant Kit, Thermo Fisher Scientific) was used to detect LPS activity in plasma samples.

### 2.6. Western Blot

The relative quantities of jejunal and colonic TJ proteins claudin-1, -2, and -4, as well as occludin, were assessed with Western Blot (WB). The tissue samples were homogenized in PBS-T (136 mM NaCl, 8 mM Na_2_HPO_4_, 2.7 mM KCl, 4.46 mM KH_2_PO_4_, 0.1% Tween, pH 7.4) containing protease inhibitor (Pierce^TM^ Protease Inhibitor Mini Tablets, Thermo Fisher Scientific) with Precellys 24 homogenizer (Bertin Technologies, Montigny le Bretonneux, France) for 3 × 20 s at 5500 rpm at 4 °C. The homogenates were sonicated for 12 s at 21% of the maximal power (VC 505 Ultrasonic Processor, Sonics, Newtown, CT, USA) and centrifuged for 15 min at 12,000× *g* at 4 °C. The protein concentrations of the supernatants were assessed with a commercial kit (Pierce^TM^ BCA Protein Assay Kit, Thermo Fisher Scientific). The supernatants were diluted to the same total protein concentration using PBS-T and Laemmli sample buffer (Bio-Rad, Hercules, CA, USA) with 5% 2-mercaptoethanol. The proteins were denatured on a heat block at 95 °C for 5 min.

Samples containing 30 μg total protein were loaded in 4–20% Mini-PROTEAN^®^ TGX^TM^ Precast gels (Bio-Rad). The gels contained 4–5 samples from each group. After the SDS-PAGE run, the proteins were transferred to a nitrocellulose membrane (Bio-Rad), which was blocked for 1 h with a commercial buffer (Odyssey blocking buffer (TBS), LI-COR, Lincoln, NE, USA) at RT and incubated in primary antibody solution overnight at 4 °C. The primary antibodies used were claudin-1 (sc-166338, 1:200; Santa Cruz Biotechnology, Dallas, TX, USA), claudin-2 (sc-293233, 1:200; Santa Cruz Biotechnology), claudin-4 (sc-376643, 1:200; Santa Cruz Biotechnology), and occludin (#91131, 1:1000, Cell Signaling Technology, Danvers, MA, USA). After washing, the membranes were incubated in fluorescence-labeled secondary antibody solution (1:10,000 (IRDye 680LT goat anti-mouse or IRDye 800CW goat anti-rabbit, LI-COR)) for 1 h at RT, protected from light. The bands were detected with the Odyssey CLx infrared imaging system (LI-COR) and analyzed with the program Image Studio (LI-COR). The protein quantities were normalized to the quantity of the loading control, β-actin (#3700, 1:3000; Cell Signaling Technology).

### 2.7. Reverse Transcription Quantitative Polymerase Chain Reaction

The mRNA expression of the TJ protein-coding genes *Cldn1*, *Cldn2*, *Cldn4*, and *Ocln* and inflammatory marker genes *Tnf*, *Il1b*, *Il6*, and *Lcn2* in jejunal and colonic tissue were analyzed with reverse transcription quantitative polymerase chain reaction (RT-qPCR). The expression of intestinal alkaline phosphatase (IAP)-subtype-coding gene *Akp6* was analyzed from jejunum. Total RNA was extracted from the tissue samples with a commercial kit (NucleoSpin RNA Kit, Macherey Nagel, Duren, Germany) and its concentration was analyzed with NanoDrop 2000 Spectrophotometer (Thermo Fisher Scientific). The samples were diluted to the same concentration, and RNA was reverse transcribed to complementary DNA with iScript^TM^ cDNA Synthesis Kit (Bio-Rad). RT-qPCR was run with LightCycler^®^ 480 SYBR Green Master (Roche Diagnostics Corp., Indianapolis, IN, USA). The amplification protocol was: 10 min at 95 °C, 40 cycles of denaturation (15 s, 95 °C), annealing (30 s, 60 °C), and elongation (30 s, 72 °C). The melt curves were analyzed at the end of the experiment. The primer sequences used are listed in Table 2.

The results were calculated as relative quantities (RQ) of messenger RNA (mRNA) normalized against the mRNA expression of three housekeeping genes according to the Vandesompele method [27]. The housekeeping genes used were *Actb*, *Eef2*, and *Rplp0.*

**Table 2:** Primer sequences used in RT-qPCR analyses. If no reference is cited, the primer pair is designed by the authors.

Gene	Forward Primer	Reverse Primer	Ref.
*Actb*	CTGAATGGCCCAGGTCTGAG	AAGTCAGTGTACAGGCCAGC	[28]
*Eef2*	TGTCAGTCATCGCCCATGTG	CATCCTTGCGAGTGTCAGTGA	[29]
*Rplp0*	TAACCCTGAAGTGCTCGACA	GGTACCCGATCTGCAGACA	[30]
*Cldn1*	AGACCTGGATTTGCATCTTGGTG	TGCAACATAGGCAGGACAAGAGTTA	[31]
*Cldn2*	GCAAACAGGCTCCGAAGATACT	GAGATGATGCCCAAGTACAGAG	[32]
*Cldn4*	TGAGCGATGGCGTCTATGG	GATGTTGCTGCCGATGAAGG	
*Ocln*	CGGTACAGCAGCAATGGTAA	CTCCCCACCTGTCGTGTAGT	[33]
*Il1b*	CTCCAGCCAAGCTTCCTTGT	TCATCACTGTCAAAAGGTGGCA	[28]
*Il6*	ATCGTGGAAATGAGAAAAGAGTTGT	CTGCAAGTGCATCATCGTTGT	
*Tnf*	TGGCACCACTAGTTGGTTGTCT	AGCCTGTAGCCCACGTCGTA	[34]
*Lcn2*	CCACCACGGACTACAACCAG	AGCTCCTTGGTTCTTCCATACAG	
*Akp6*	ACCGAAGCTCAGAGTGTTGAT	GCAAATATGGCCACGTCCTC	

### 2.8. Intestinal Alkaline Phosphatase Activity

Fecal pellets were suspended in alkaline phosphatase extraction buffer (10 mM Tris-HCl, 1 mM MgCl_2_, 0.1 mM ZnCl_2_, pH 8.0) containing protease inhibitor (cOmplete™, EDTA-free Protease Inhibitor Cocktail, Roche Diagnostics Corp.) with a pipette tip, followed by vortexing for 10 min. Samples were centrifuged for 5 min at 5000× *g* at 4 °C for the removal of insoluble matrix, after which the supernatants were transferred into new tubes and centrifuged for 15 min at 12,000× *g* at 4 °C.

IAP activity was assayed from the supernatants using a *p*-nitrophenyl phosphate (pNPP)-based assay. A standard curve was generated for the assay by incubating known amounts of pNPP (Sigma-Aldrich, St. Louis, MO, USA) with commercial calf IAP. All dilutions were made in assay buffer (10 mM Tris-HCl, 1 mM MgCl_2_, 0.1 mM ZnCl_2_, pH 10). The samples were incubated in 0.45 mM pNPP solution for 30 min at 37 °C. The absorbances were read at 405 nm and the amount of formed *p*-nitrophenol (pNP) was read from the standard curve. IAP activity was calculated as pNP formed in µmol*min^−1^ per g of protein (units per g) in the sample. Protein concentrations were assayed using the Pierce^TM^ BCA Protein Assay Kit (Thermo Fisher Scientific).

### 2.9. Statistical Analyses

Statistical analyses were conducted, and figures drawn with GrapPad Prism 8 (Dotmatics, La Jolla, CA, USA). The level of statistical significance was set at *p* < 0.05. Permeability to iohexol was analyzed using mixed-effects model followed by Sidak’s post-hoc test. Based on normal distribution determined by Shapiro–Wilk test, other data were analyzed either using one-way ANOVA followed by Tukey’s post-hoc test, or Kruskal–Wallis followed by Dunn’s post-hoc test. Unless stated otherwise, data are presented as mean except for RT-qPCR results that are expressed as geometric mean.

## 3. Results

### 3.1. Metabolic Parameters

**Figure 2:**
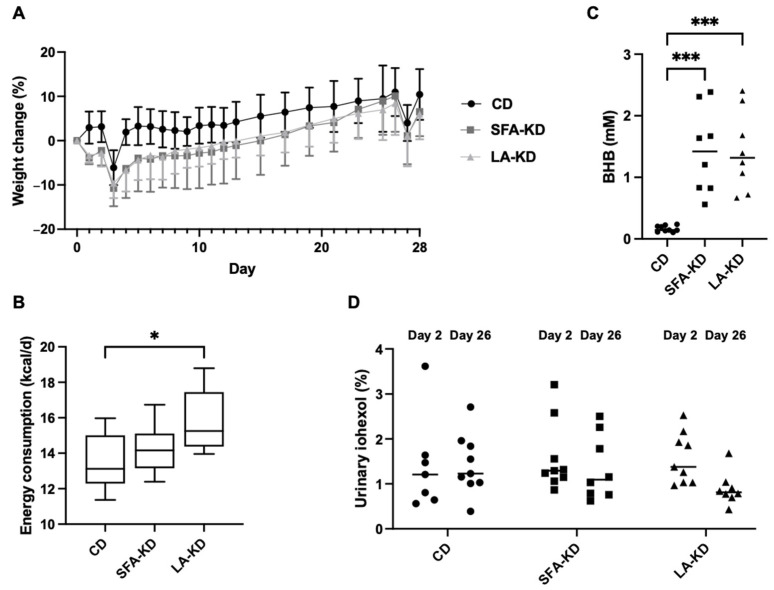
Metabolic parameters and intestinal permeability to iohexol. (**A**). Body weight change as a percentage from the baseline (mean ± SD, *n* = 8–10/group). (**B**). Average energy consumption (mean ± SD, *n* = 8–10 per group) showing higher energy consumption in LA-KD. (**C**). Plasma BHB levels showing elevated levels in KD groups. (**D**). Intestinal permeability measured as the percentage of iohexol in the urine after 24-h collection showing no between- or within-group differences. * *p* < 0.05, *** *p* < 0.001. BHB = β-hydroxybutyrate, CD = control diet, LA-KD = ketogenic diet with linoleic acid, SFA-KD = ketogenic diet with saturated fatty acids.

At the start of the experiment, there were no significant differences in body weights between the three groups. Initially, animals in both KD groups lost weight but fully regained it after two weeks on the diet (Figure 2A). From that point onwards, the weight changes followed the same trajectory as controls. All groups lost weight upon metabolic caging (days two and 26) but regained it within two days after being returned to home cages. 

The average energy consumption in LA-KD was slightly higher than in CD despite no differences in weight gain (Figure 2B). Energy intake in SFA-KD did not differ from other groups. The initial loss of body weight seen in both KD groups was not related to lower energy intake. The effect of metabolic caging was reflected in energy consumption, which was lower on the days of these experiments.

We analyzed plasma BHB to confirm the level of nutritional ketosis. Generally, the state of ketosis is defined as plasma BHB level of 0.5 mM or above [35]. As expected, all animals on KDs showed elevated BHB levels with no difference between the two groups (Figure 2C). Animals on CD were not in ketosis.

### 3.2. Intestinal Permeability

#### 3.2.1. Permeability to Iohexol

On day two and day twenty=six of the experiment, we orally administered iohexol to the animals to compare paracellular permeability of the intestine between groups. There were no significant between-group differences in the percentual 24-h urinary recovery of administered iohexol after two or twenty-six days of dietary intervention (Figure 2D), indicating that intestinal permeability to this compound was not altered at either time point. While there was no statistical change within groups between two and twenty-six days of feeding, a non-significant (*p* = 0.097) decrease of 43% was observed in LA-KD.

#### 3.2.2. Tight Junctions

**Figure 3:**
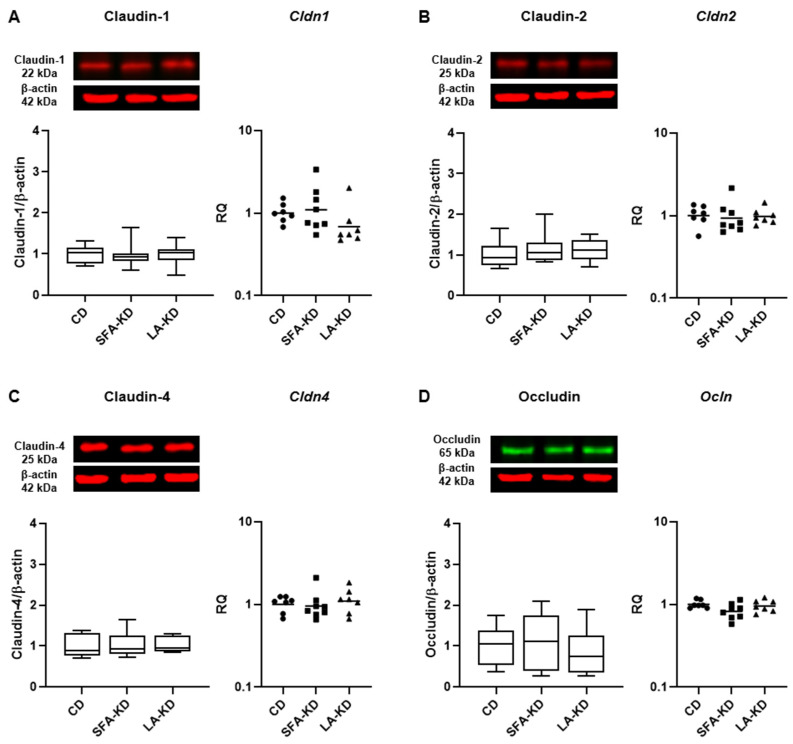
Jejunal tight junction protein and mRNA expressions of (**A**). claudin-1, (**B**). claudin-2, (**C**). claudin-4, and (**D**). occludin analyzed with Western Blot (*n* = 7–9) and RT-qPCR showing no differences in the expression of these proteins. Images of original blots are presented in Figure S1. CD = control diet, LA-KD = ketogenic diet with linoleic acid, RQ = relative quantity, SFA-KD = ketogenic diet with saturated fatty acids.

**Figure 4:**
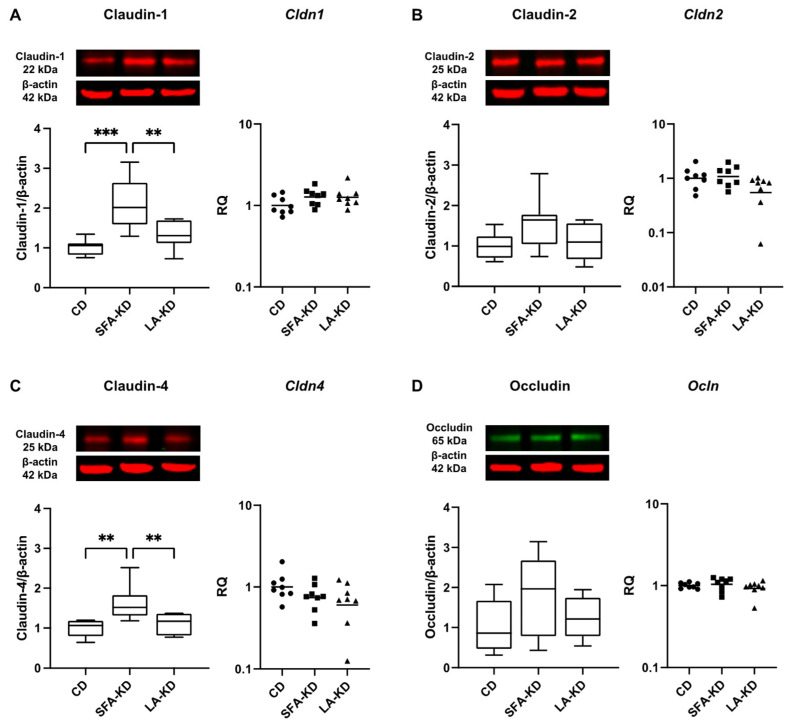
Colonic tight junction protein and mRNA expressions of (**A**). claudin-1, (**B**). claudin-2, (**C**). claudin-4, and (**D**). occludin analyzed with Western Blot (*n* = 7–9) and RT-qPCR showing increased protein expression of claudin-1 and -4 in SFA-KD. Images of original blots are presented in Figure S2. ** *p* < 0.01, *** *p* < 0.001. CD = control diet, LA-KD = ketogenic diet with linoleic acid, RQ = relative quantity, SFA-KD = ketogenic diet with saturated fatty acids.

To investigate other changes in the epithelial barrier, we assessed the relative levels of TJ proteins claudin-1, -2, and -4, and occludin with WB and the expression of TJ protein-coding genes determined with RT-qPCR. While there were no differences in jejunum (Figure 3), the analyses revealed a statistically significant increase in the colonic expression of the barrier-sealing TJ proteins claudin-1 and claudin-4 in SFA-KD in comparison to CD and LA-KD (Figure 4). The relative level of claudin-1 was doubled in SFA-KD when compared to CD and was 57% higher in comparison with LA-KD. The expression of claudin-4 in SFA-KD was 62% higher with comparison to CD and 50% higher than in LA-KD. Levels of claudin-2 and occludin were not statistically different between groups in either part of the intestine. The mRNA expressions of *Cldn1*, *Cldn2*, *Cldn4*, and *Ocln* were not different between groups in either jejunum or colon (Figure 3 and Figure 4).

### 3.3. Epithelial Cell Vacuolation

**Figure 5:**
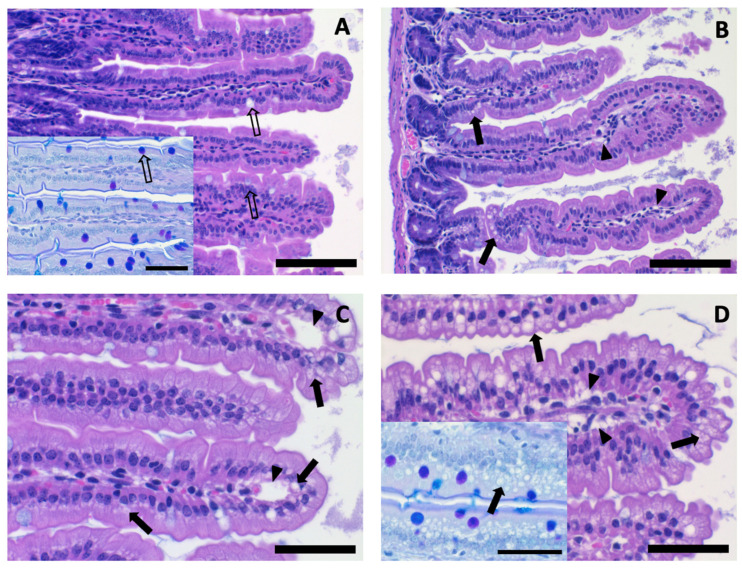
Representative microphotographs of HE- and AB-PAS-stained (insets) sections of jejunal villi. (**A**). No epithelial vacuolation in CD. Goblet cells (open arrows). Bar 100 µm, 20× objective magnification. Inset: Close-up of an AB-PAS-stained section. Goblet cell mucins stain bluish with AB and or purple with PAS. Bar 50 µm, 40× obj. mag. (**B**). Grade 1 vacuolation in LA-KD. Single small, clear apical vacuoles (arrows) present in the epithelial cells in the basal half of the villi and slight subepithelial edema (arrowheads). Bar 100 µm, 40× obj. mag. (**C**). Grade 2 vacuolation in LA-KD. Moderate vacuolation (arrows) partly occupying most epithelial cells and extending to villus tips that also show subepithelial blebs (arrowheads; artefactual change accentuated by edema). Bar 50 µm, 40× obj. mag. (**D**). Grade 3 vacuolation in SFA-KD. Marked vacuolation (arrows) present throughout the villi and extensively occupying epithelial cell cytoplasms, and subepithelial edema (arrowheads). Bar 50 µm, 40× obj. mag. Inset: Close-up of an AB-PAS-stained section. The epithelial vacuoles are AB-PAS negative. Bar 50 µm, 40× obj. mag. AB = Alcian blue, CD = control diet, HE = hematoxylin and eosin, LA-KD = ketogenic diet with linoleic acid, PAS = Periodic acid–Schiff, SFA-KD = ketogenic diet with saturated fatty acids.

Jejunum and colon samples showed no definite histological lesions. However, jejunal villus epithelial cells in both KD groups exhibited vacuolation, which was more pronounced in SFA-KD than in LA-KD (Figure 5). There was no vacuolation in the colon. The extent of jejunal vacuolation varied from basal 1/3 of the villus length to diffuse involvement, and the intensity from single vacuoles in apical cytoplasm to foaminess and/or marked vacuolation throughout the cell. In addition, minimal to mild villous subepithelial edema, manifested as sparse lamina propria and/or focal detachment of the epithelial cells from the underlying lamina propria in sporadic villi, was present in SFA-KD and LA-KD mice (Figure 5). The CD mice showed no epithelial vacuolation (EV grade = 0) and only one animal exhibited villous edema. In contrast, in LA-KD the average EV grade was 1.8 and 6 of the 8 samples analyzed showed villus edema, and in SFA-KD the average EV grade was 2.6 and villus edema was present in 8 of 9 samples. The vacuoles did not contain simple polysaccharides or mucins as shown by the AB-PAS staining, and no vacuolation or edema was observed in colon, consistent with the hypothesis that jejunal vacuoles resulted from absorbed dietary fat.

### 3.4. Inflammation

**Figure 6:**
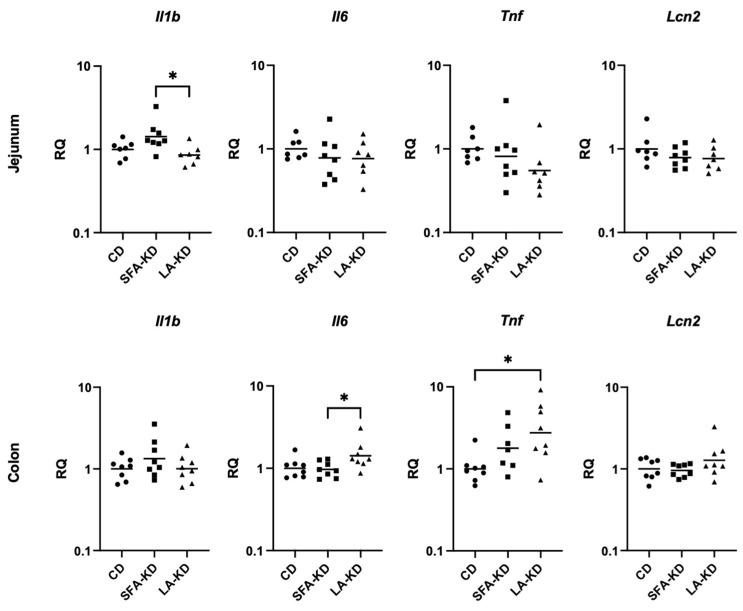
Jejunal and colonic mRNA expressions of inflammatory markers. SFA-KD promoted the expression of *Il1b* in jejunum and LA-KD *Il6* and *Tnf* expression in jejunum. * *p* < 0.05. CD = control diet, LA-KD = ketogenic diet with linoleic acid, RQ = relative quantity, SFA-KD = ketogenic diet with saturated fatty acids.

We used RT-qPCR to analyze the gene expression levels of the markers of inflammation *Tnf*, *Il1b*, *Il6*, and *Lcn2* in the intestine. Small but statistically significant changes were detected: in jejunum, *Il1b* mRNA levels were slightly higher in SFA-KD than in LA-KD without differences when compared to CD (Figure 6), while in colon, LA-KD promoted the expression of *Tnf* relative to CD and *Il6* to SFA-KD.

### 3.5. Intestinal Alkaline Phosphatase

**Figure 7:**
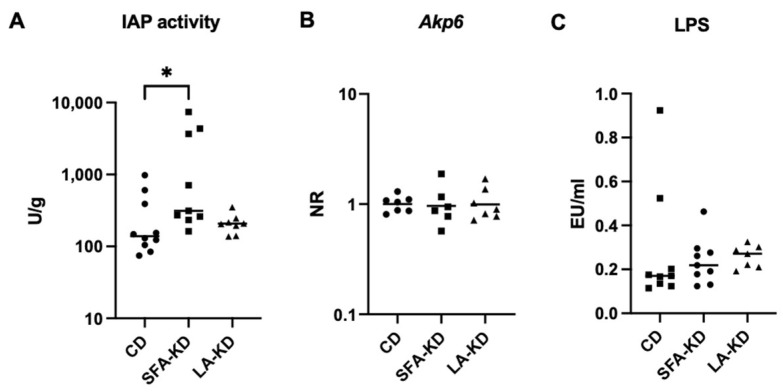
IAP activity, *Akp6* expression and LPS. (**A**) IAP activity (U/g) measured from feces showing increased activity in SFA-KD. Data is presented as median. (**B**) *Akp6* expression in jejunum showing no differences. (**C**) LPS activity (EU/mL) in plasma showing no differences. Data is presented as median. * *p* < 0.05. CD = control diet, IAP = intestinal alkaline phosphatase, LA-KD = ketogenic diet with linoleic acid, LPS = lipopolysaccharide, RQ = relative quantity, SFA-KD = ketogenic diet with saturated fatty acids.

We examined the activity of IAP, an anti-inflammatory and TJ protein level-promoting enzyme, in the feces in order to explore potential contributors to the increased TJ protein expression. Activity of the enzyme was increased in SFA-KD but not in LA-KD when compared to CD (Figure 7A). The activity between the two KD groups did not differ significantly.

Due to the increased IAP activity in feces, we also analyzed the jejunal expression of *Akp6*, which encodes for a mouse-specific IAP subtype evenly expressed throughout the intestine. However, increased activity did not correspond to changes in mRNA expression in the tissue (Figure 7B).

### 3.6. Lipopolysaccharide

In addition to markers of paracellular permeability, we investigated whether the study diets influence LPS translocation from the gut lumen to circulation. Plasma LPS activity, however, was not significantly increased in either of the KD groups (Figure 7C). Removal of outliers did not change the result.

## 4. Discussion

The aim of this study was to investigate whether ketogenic diets (KDs) with disparate fat sources impact paracellular permeability or cause inflammation in the intestine differently. We fed healthy mice with two KDs—one group a diet high in saturated fatty acids SFAs from milkfat (SFA-KD) and the other group a diet abundant in linoleic acid from vegetable sources (LA-KD). Intestinal permeability to iohexol was measured at two time points: after two and twenty-six days from the start of the dietary intervention. As other parameters related to permeability, we analyzed jejunal and colonic TJ protein expression, histological changes, and inflammatory markers, as well as changes in the activity and expression of IAP. Moreover, LPS activity levels were determined in plasma samples. KDs induced small changes in intestinal TJ proteins, inflammatory parameters, and fecal IAP activity, without a significant impact on paracellular permeability to iohexol.

We determined intestinal permeability to iohexol, which is a stable, low-molecular weight (821 Da) compound validated for the assessment of paracellular permeability of the whole intestine in mice [36]. As a marker of permeability, iohexol has been compared to ^51^Cr-EDTA with high correlation between the two [37], and permeability to iohexol is a superior predictor of disease activity in inflammatory bowel disease patients when compared to lactulose-mannitol ratio [38]. In our study, permeability to iohexol differed neither between groups at either time point nor within groups between time points. Mice enter ketosis after 24–48 h of severe carbohydrate restriction [39]. Here we show that short-term (two days) or longer-term (four-week) feeding with KD does not influence paracellular permeability to iohexol and, thus, it does not seem to change upon the shift from carbohydrate-based to ketone-based metabolism or as a result of four weeks of ketosis. These results illustrate that while some studies have shown barrier function to be impaired by HFD for durations ranging from one to fifteen weeks [6,7,40] or specific fats fed for eight weeks [23], the same does not seem to apply in the context of four-week KD in mice. Our findings are also in line with the observation that even though a low-fiber HFD can lead to the bacterial degradation of the colonic mucus layer in mice [41], this does not happen with high-fat KDs despite the lack of fermentable fiber [15]. KD is distinctly different from HFDs regularly used in animal experiments due to its lack of carbohydrates and the resulting state of ketosis. It is possible that the effects of dietary fat and its source on paracellular permeability may differ depending on the composition of the diet as a whole.

Despite no differences in permeability, we observed SFA-KD but not LA-KD to promote the colonic expression of TJ proteins claudin-1 and claudin-4 on protein but not on mRNA level. Claudins comprise a family of 27 known adhesion proteins, with key roles in the paracellular barrier and channel functions of TJs [42]. While we saw no changes in jejunal TJ protein expression, others have reported KD to increase the expression of small intestinal claudin-1 protein but not gene expression in conjunction with lowering inflammation and restoring crypt length in a rat model of irritable bowel syndrome [17]. Even though these claudins are considered to be barrier-sealing [43,44], in human biopsies their expression is elevated in active inflammatory bowel disease, and claudin-1 levels correlate with inflammatory activity [45]. In addition, claudin-1 overexpression results in greater susceptibility to and poorer recovery from DSS-induced colitis [46], making it unclear whether the increase in claudin-1 in our study is a beneficial change or a compensatory mechanism.

KDs influenced the gene expression of inflammatory markers *Il1b*, *Il6*, and *Tnf* in the intestine. Intriguingly, jejunal *Il1b* levels were elevated in SFA-KD when compared to LA-KD, but colonic *Il6* was upregulated in LA-KD in comparison to SFA-KD and *Tnf* in comparison to CD. While statistically significant, these between-group differences were small, bringing into question their physiological meaning. Nevertheless, it is possible that upon assault, these changes might become of importance. Based on previous studies, high LA in the context of a diet rich in both fat and carbohydrate does not increase permeability [23] but might compromise the intestinal barrier upon exposure to LPS [21] or alcohol [22]. KDs have been studied as an intervention for DSS-induced experimental colitis, but the results are contradictory—one group reported KD to ameliorate colonic inflammation [19], whereas another noted the diet to worsen it [20]. While there are several differences between these studies, one contributing factor might be the fat source of the diet. Here, we saw that SFA- and LA-rich KDs had dissimilar impacts on both small and large intestine and the differences might become more pronounced upon induced intestinal inflammation. Since we only observed subtle differences in the intestine, plasma levels of these markers were not assessed.

Triglyceride consumption has been shown to induce lipid droplet and chylomicron formation in enterocytes in various species, including mice, with well-known mechanisms [47,48]. However, to our knowledge, we are the first to report high-fat KDs to result in histologically detectable increased, in all probability lipid, vacuolation in jejunal enterocytes and minimal to mild subepithelial villous edema. While investigating the nature of the vacuoles in depth was outside the scope of this study, we found them to be AB-PAS negative, excluding polysaccharide or mucin content, which corroborates the lipid presumption and is logical in the context of a diet high in fat. We propose that the observed histological findings indicate an adaptive response and might be a natural consequence of drastically increased fat intake in a species adapted to eat a low-fat diet. The enterocyte vacuoles and foaminess suggestively denote the extremely high number of enterocyte lipid droplets acting as triacylglycerol storage pool and the chylomicrons, and the subepithelial edema the chylomicron flow towards villus lacteals (see [49,50] for recent reviews). Consistent with the modest protein and mRNA expression changes, we detected no further histological alterations in the jejunum and no histological changes in the colon. Long-term HFDs from eight to fourteen weeks of length have been reported to shorten villus length and crypt depth as well as to reduce goblet cell numbers [50]. The number of vacuoles was markedly higher in SFA-KD than in LA-KD. This could be explained by the finding that at least in Caco-2 cells, LA is more potent than SFAs in stimulating the synthesis of triglycerides and secretion of chylomicrons [51,52], and thus, is likely to be more efficiently cleared from the cells, albeit in in vivo studies such differences have not been reported [49,50].

A novel finding was that a KD high in SFAs promotes the fecal activity of IAP, despite not changing its expression on a transcriptional level in jejunum. This enzyme produced by enterocytes is generally recognized to be beneficial for intestinal health for its anti-inflammatory, LPS-detoxifying properties [53]. Importantly, IAP influences the expression of TJ proteins, and has specifically been shown to upregulate claudin-1 mRNA levels in a mouse model of sepsis [54]. The increase we observed in the expression of claudins, especially claudin-1, could be partly a result of the higher activity of IAP. HFDs and omega-3 fatty acids have been shown to promote the expression and activity of IAP [55]. With the former, this is thought to be a response against increased diet-induced LPS translocation and resulting endotoxemia, whereas omega-3s upregulate IAP production through different mechanisms, such as the production of inflammation-resolving resolvins [56]. SFAs in the context of a regular diet can increase LPS transport, especially when derived from palm or coconut oil [26]. However, we did not observe higher plasma LPS activity as a response to a SFA-rich KD with milkfat as the fat source nor to a LA-rich KD, indicating that LPS translocation might not increase as a response to KD similarly to a non-ketogenic HFD [7]. This could also be a result of upregulated detoxification or an effect of the fat source since milk-derived phospholipids seem to protect from HFD-induced increases in LPS-binding protein [57]. The ketone body BHB has been reported to increase IAP expression in vitro in both LS174T and HT29 cell lines and in vivo in mouse small intestine [58]. Thus, increased BHB levels might also be an explanatory factor for the higher activity in SFA-KD. However, while SFA-KD promoted IAP activity, LA-KD did not. Interestingly, DeCoffe et al. [59] observed no differences in IAP activity between diets containing 20% SFA or corn oil, high in LA, after infecting rats with *Citrobacter rodentium*. The addition of 1% of fish oil to the diet increased the activity and provided protection from the infection when given with SFA but had detrimental effects with the high-LA diet. Thus, it is possible that SFAs paired with an anti-inflammatory agent can promote IAP activity and, in our setting, the combination of SFAs and BHB, a molecule shown to attenuate inflammation [60], could function similarly. In line with the results of DeCoffe et al., a LA-rich diet does not seem to have the same effect.

The systemic effects of the diets, except for plasma LPS activity, were not included in the frame of this study since mice are poor models for cardiovascular effects of HFDs. Dietary fat quality and quantity are generally recognized to influence cardiovascular disease, and while KDs have been studied in the context of cardiovascular disease risk (see [61] for a recent review), the research on the effects of the fat source of these diets is scarce. Since we did not assess the systemic effects of these diets, predicting the cardiovascular influence of the diets used was not possible. It also remains to be investigated whether other fat sources, such as those high in monounsaturated fat, influence barrier function, inflammation, and IAP activity in the intestine differently in the context of KD. The challenge with this is diet formulation; KDs for rodents require a solid base which makes studying liquid oils, such as olive oil, difficult in this setting. In addition, it is not possible to isolate the effects of specific fatty acids with these diets. Most fat sources are comprised of a variety of different fatty acids and thus definitive conclusions on their effects cannot be drawn. On the other hand, human diets—whether ketogenic or not—usually contain fat from a variety of sources, which might either counteract or enhance each other’s effects.

## 5. Conclusions

Our study indicates that KDs, regardless of fat source, do not directly influence intestinal permeability to iohexol in healthy mice after two days or four weeks of feeding. However, we saw KDs to cause modest changes in indirect measures related to intestinal permeability, altering colonic TJ protein levels and cytokine expression in both jejunum and colon. In addition, we detected increased IAP activity and jejunal vacuolation as a response to KD high in SFAs. Future research is warranted to show whether these changes become of significance in a proinflammatory setting.

## Figures and Tables

**Table 1:** The macronutrient compositions of the diets. Values are presented as percentage of total energy. CD = control diet, SFA-KD = ketogenic diet with saturated fatty acids, LA-KD = ketogenic diet with linoleic acid.

	CD	SFA-KD	LA-KD
Protein (E%)	9.7	9.4	9.4
Carbohydrate (E%)	77.8	0.5	0.5
Fat (E%)	12.5	90.1	90.1
Saturated fat (E%)	2.6	56.6	22.8
Monounsaturated fat (E%)	3.0	26.0	17.9
Polyunsaturated fat (E%)	6.9	4.5	49.3
Linoleic acid (E%)	6.4	4.0	44.5

## Data Availability

The data used in this study are available from corresponding author upon reasonable request.

## Supplementary Materials

The following supporting information can be downloaded at: https://www.mdpi.com/article/10.3390/nu16010018/s1, Figure S1: Original and unmodified Western Blot images of jejunal tight junction proteins. Figure S2: Original and unmodified Western Blot images of colonic tight junction proteins.
