# Correction: Toivio et al. Ketogenic Diet High in Saturated Fat Promotes Colonic Claudin Expression without Changes in Intestinal Permeability to Iohexol in Healthy Mice. *Nutrients* 2024, *16*, 18

**DOI:** 10.3390/nu16203471

**Published:** 2024-10-14

**Authors:** Lotta Toivio, Hanna Launonen, Jere Lindén, Markku Lehto, Heikki Vapaatalo, Hanne Salmenkari, Riitta Korpela

**Affiliations:** 1 Department of Pharmacology, Faculty of Medicine, University of Helsinki, 00014 Helsinki, Finland; hanna.launonen@helsinki.fi (H.L.); heikki.vapaatalo@helsinki.fi (H.V.); 2 Human Microbiome Research Program, Faculty of Medicine, University of Helsinki, 00014 Helsinki, Finland; 3 Department of Veterinary Biosciences, Faculty of Veterinary Medicine, University of Helsinki, 00014 Helsinki, Finland; jere.linden@helsinki.fi; 4 Finnish Centre for Laboratory Animal Pathology, Helsinki Institute of Life Science, University of Helsinki, 00014 Helsinki, Finland; 5 Folkhälsan Institute of Genetics, Folkhälsan Research Center, 00290 Helsinki, Finland; markku.lehto@helsinki.fi (M.L.); hanne.salmenkari@helsinki.fi (H.S.); 6 Department of Nephrology, University of Helsinki and Helsinki University Hospital, 00290 Helsinki, Finland; 7 Research Program for Clinical and Molecular Metabolism, Faculty of Medicine, University of Helsinki, 00014 Helsinki, Finland

**Text Correction**

There was an error in the original publication [1] in the name of the company Thermo Fisher Scientific. Corrections have been made to Section 2.4, Paragraph 1; Section 2.5; Section 2.8, Paragraph 2.

“Tissue pieces from jejunum (opened and unopened) and colon (unopened) were fixed in 4% buffered paraformaldehyde (Thermo Fisher Scientific, Waltham, MA, USA) solution for 36 h and then stored at 4 °C in 70% ethanol. The opened jejunal samples were cut into longitudinal halves, and the unopened jejunal parts and colon samples trimmed transversally. The fixed samples were dehydrated, embedded in paraffin, and cut into 4 μm thick sections. All sections were stained with hematoxylin and eosin (HE) stain, and selected jejunal sections with Alcian blue–Periodic Acid–Schiff (AB-PAS) stain, which detects intestinal mucins (goblet cells) and polysaccharides.”

“The state of ketosis was confirmed by measuring plasma BHB levels with a commercial enzymatic kit (Cayman Chemicals, Ann Arbor, MI, USA). A Limulus Amebocyte Lysate Assay (Pierce^TM^ Chromogenic Endotoxin Quant Kit, Thermo Fisher Scientific) was used to detect LPS activity in plasma samples.”

“IAP activity was assayed from the supernatants using a *p*-nitrophenyl phosphate (pNPP)-based assay. A standard curve was generated for the assay by incubating known amounts of pNPP (Sigma-Aldrich, St. Louis, MO, USA) with commercial calf IAP. All dilutions were made in assay buffer (10 mM Tris-HCl, 1 mM MgCl_2_, 0.1 mM ZnCl_2_, pH 10). The samples were incubated in 0.45 mM pNPP solution for 30 min at 37 °C. The absorbances were read at 405 nm and the amount of formed *p*-nitrophenol (pNP) was read from the standard curve. IAP activity was calculated as pNP formed in µmol*min^−1^ per g of protein (units per g) in the sample. Protein concentrations were assayed using the Pierce^TM^ BCA Protein Assay Kit (Thermo Fisher Scientific).”

There was another error in the recipe for PBS-T, where the concentration of Tween was stated to be 0.001%. A correction has been made to Section 2.6, Paragraph 1. In the same paragraph, name of the company Thermo Fisher has been corrected. 

“The relative quantities of jejunal and colonic TJ proteins claudin-1, -2, and -4, as well as occludin, were assessed with Western Blot (WB). The tissue samples were homogenized in PBS-T (136 mM NaCl, 8 mM Na_2_HPO_4_, 2.7 mM KCl, 4.46 mM KH_2_PO_4_, 0.1% Tween, pH 7.4) containing protease inhibitor (Pierce^TM^ Protease Inhibitor Mini Tablets, Thermo Fisher Scientific) with Precellys 24 homogenizer (Bertin Technologies, Montigny le Bretonneux, France) for 3 × 20 s at 5500 rpm at 4 °C. The homogenates were sonicated for 12 s at 21% of the maximal power (VC 505 Ultrasonic Processor, Sonics, Newtown, CT, USA) and centrifuged for 15 min at 12,000× *g* at 4 °C. The protein concentrations of the supernatants were assessed with a commercial kit (Pierce^TM^ BCA Protein Assay Kit, Thermo Fisher Scientific). The supernatants were diluted to the same total protein concentration using PBS-T and Laemmli sample buffer (Bio-Rad, Hercules, CA, USA) with 5% 2-mercaptoethanol. The proteins were denatured on a heat block at 95 °C for 5 min.”

There was also an error in the dilution of occludin antibody which was stated to be 1:10,000. A correction has been made to Section 2.6, Paragraph 2.

“Samples containing 30 μg total protein were loaded in 4–20% Mini-PROTEAN^®^ TGX^TM^ Precast gels (Bio-Rad). The gels contained 4–5 samples from each group. After the SDS-PAGE run, the proteins were transferred to a nitrocellulose membrane (Bio-Rad), which was blocked for 1 h with a commercial buffer (Odyssey blocking buffer (TBS), LI-COR, Lincoln, NE, USA) at RT and incubated in primary antibody solution overnight at 4 °C. The primary antibodies used were claudin-1 (sc-166338, 1:200; Santa Cruz Biotechnology, Dallas, TX, USA), claudin-2 (sc-293233, 1:200; Santa Cruz Biotechnology), claudin-4 (sc-376643, 1:200; Santa Cruz Biotechnology), and occludin (#91131, 1:1000, Cell Signaling Technology, Danvers, MA, USA). After washing, the membranes were incubated in fluorescence-labeled secondary antibody solution (1:10,000 (IRDye 680LT goat anti-mouse or IRDye 800CW goat anti-rabbit, LI-COR)) for 1 h at RT, protected from light. The bands were detected with the Odyssey CLx infrared imaging system (LI-COR) and analyzed with the program Image Studio (LI-COR). The protein quantities were normalized to the quantity of the loading control, β-actin (#3700, 1:3000; Cell Signaling Technology).”

Finally, there was a typographical error for the city Karlslunde which was misspelled as “Karslunde”. A correction has been made to Section 2.1, Paragraph 1.

“The study was approved by the animal research board of the Regional State Administrative Agency for Southern Finland (ESAVI/9377/2019). Seven-week-old male C57BL/6J mice (*n* = 28) were purchased from Scanbur (Karlslunde, Denmark). The study began after 17 days of acclimatization when the animals were nine weeks old. They were kept under a 12 h light–dark cycle at 20 ± 2 °C and 50–60% humidity with *ad libitum* access to food and water.”

In addition, the corresponding author Lotta Toivio updated her email as well. The authors state that the scientific conclusions are unaffected. This correction was approved by the Academic Editor. The original publication has also been updated.

## References

[1] Toivio L., Launonen H., Lindén J., Lehto M., Vapaatalo H., Salmenkari H., Korpela R. (2024). Ketogenic Diet High in Saturated Fat Promotes Colonic Claudin Expression without Changes in Intestinal Permeability to Iohexol in Healthy Mice. Nutrients.

