# Supplementary material for: Ketogenic Diet High in Saturated Fat Promotes Colonic Claudin Expression without Changes in Intestinal Permeability to Iohexol in Healthy Mice
Source: Nutrients. 2023 Dec 20;16(1):18. doi: 10.3390/nu16010018 (PMC10780785; doi:10.3390/nu16010018)
Supplement: Supplementary file 1 [file nutrients-16-00018-s001.zip › nutrients-2682706-supplementary.pdf]

Supplementary material

A

Claudin-1

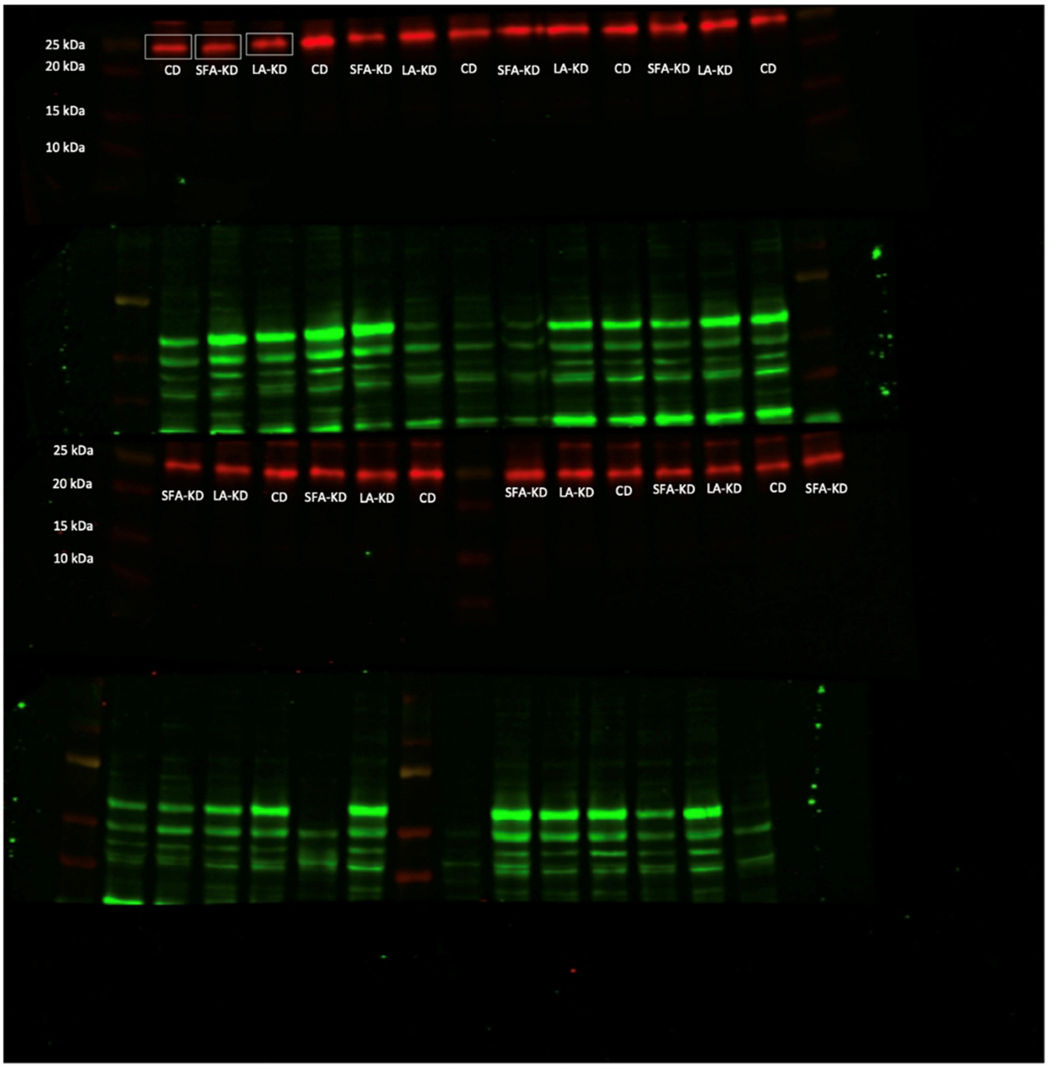

β-actin for claudin-1

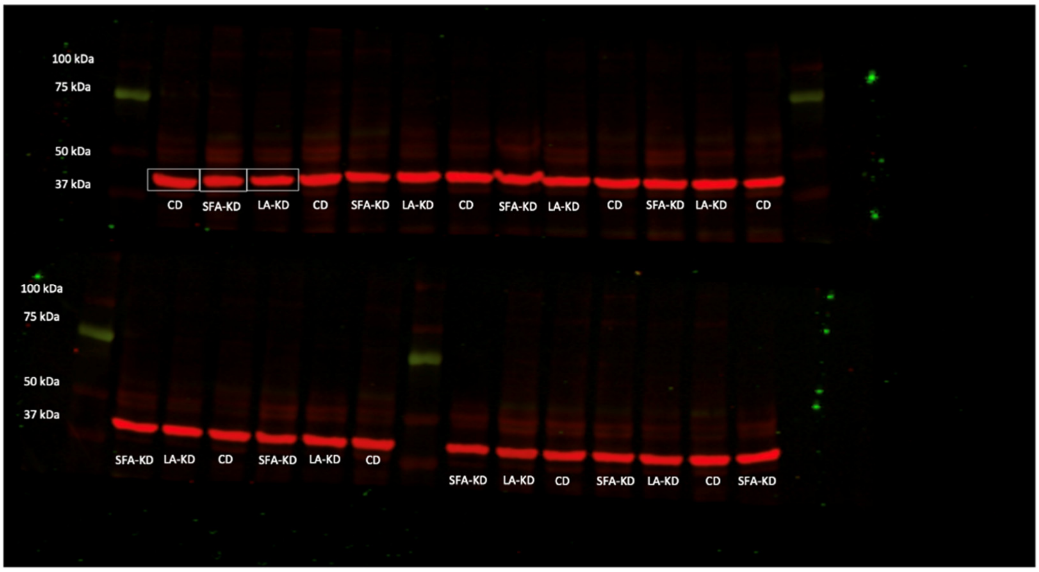

B

Claudin-2

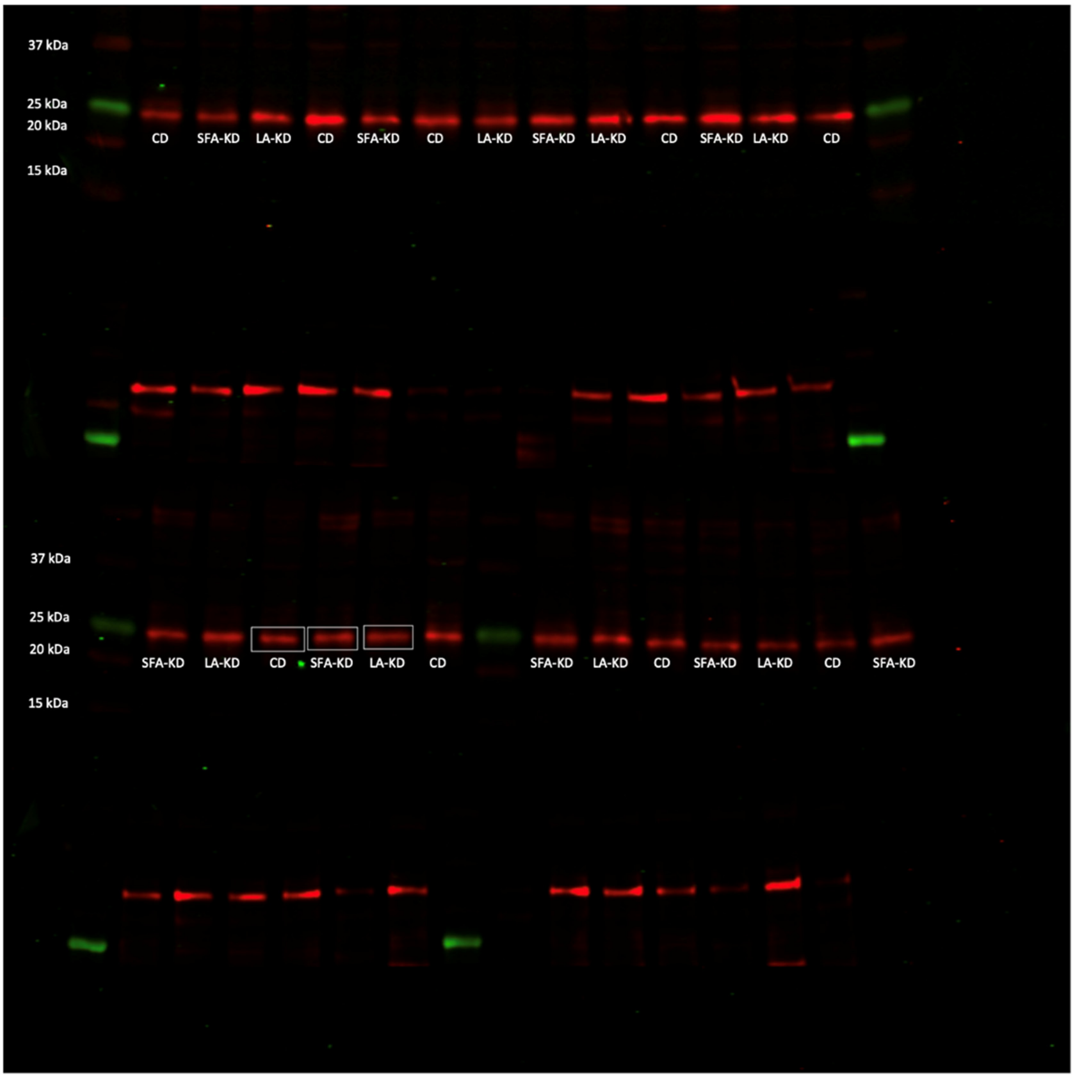

β-actin for claudin-2

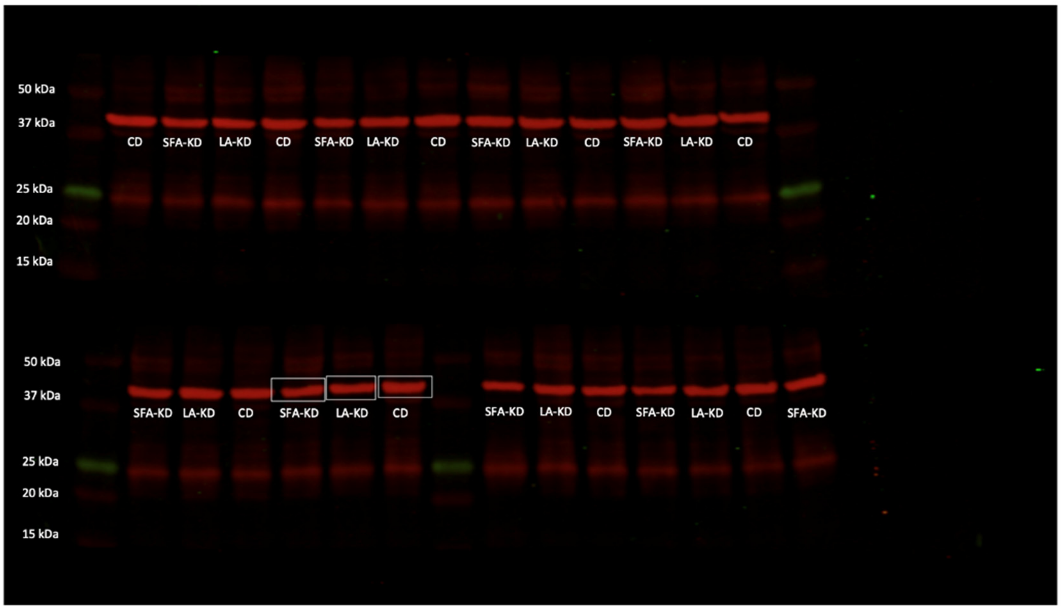

C **Claudin-4 and β-actin**

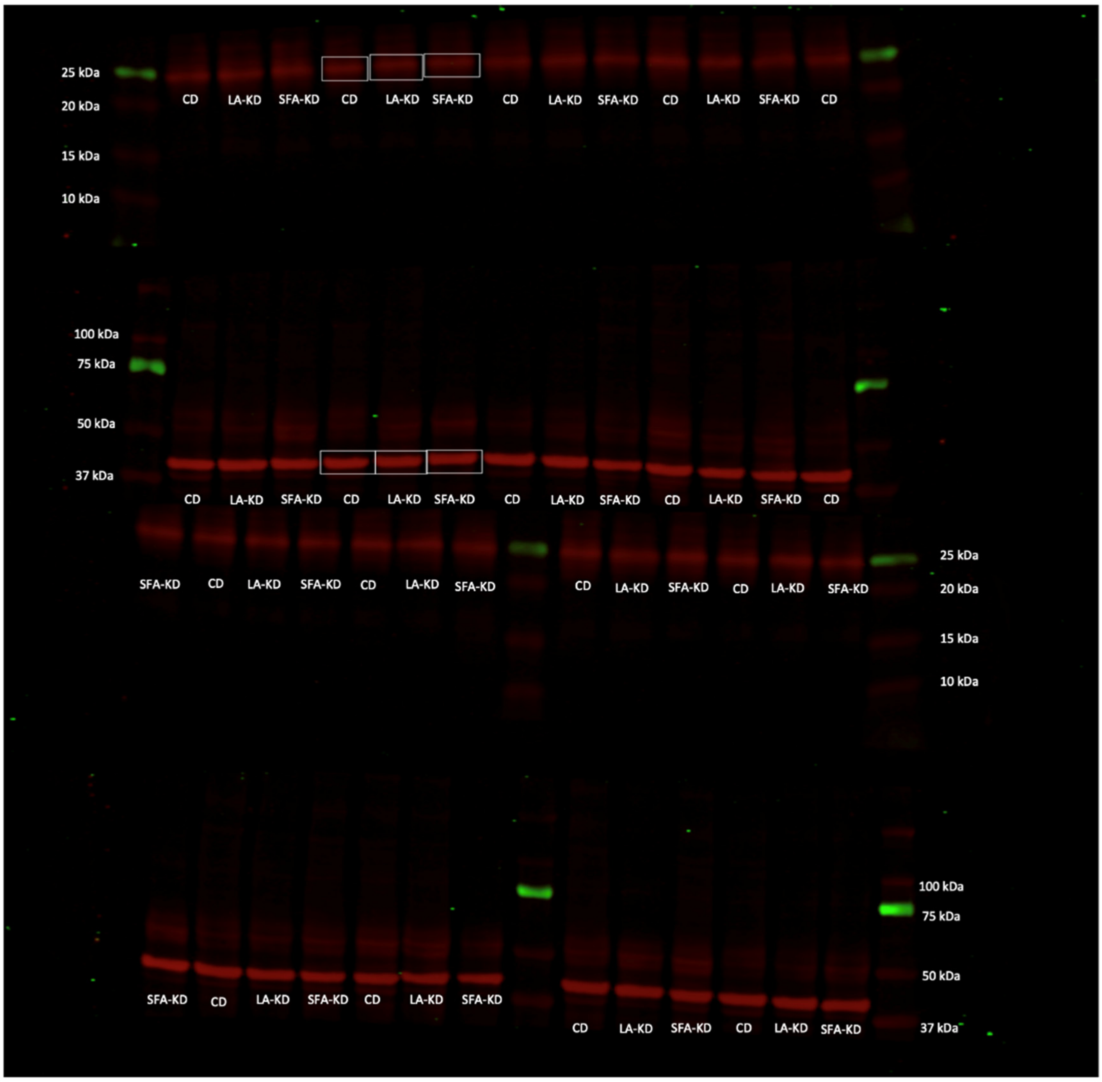

D **Occludin**

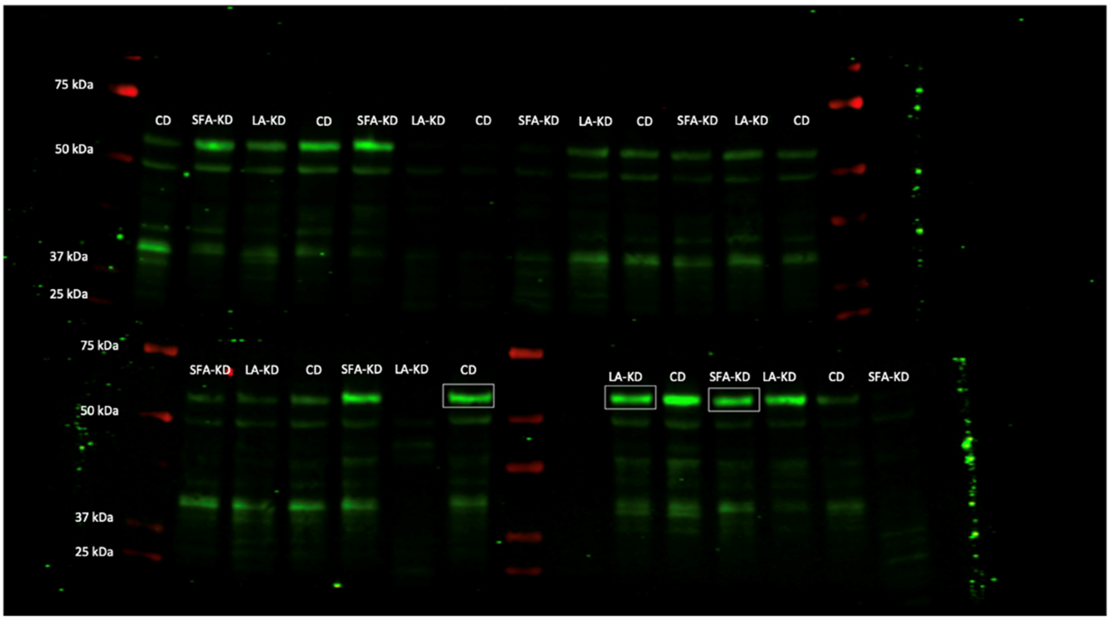

β-actin for occludin

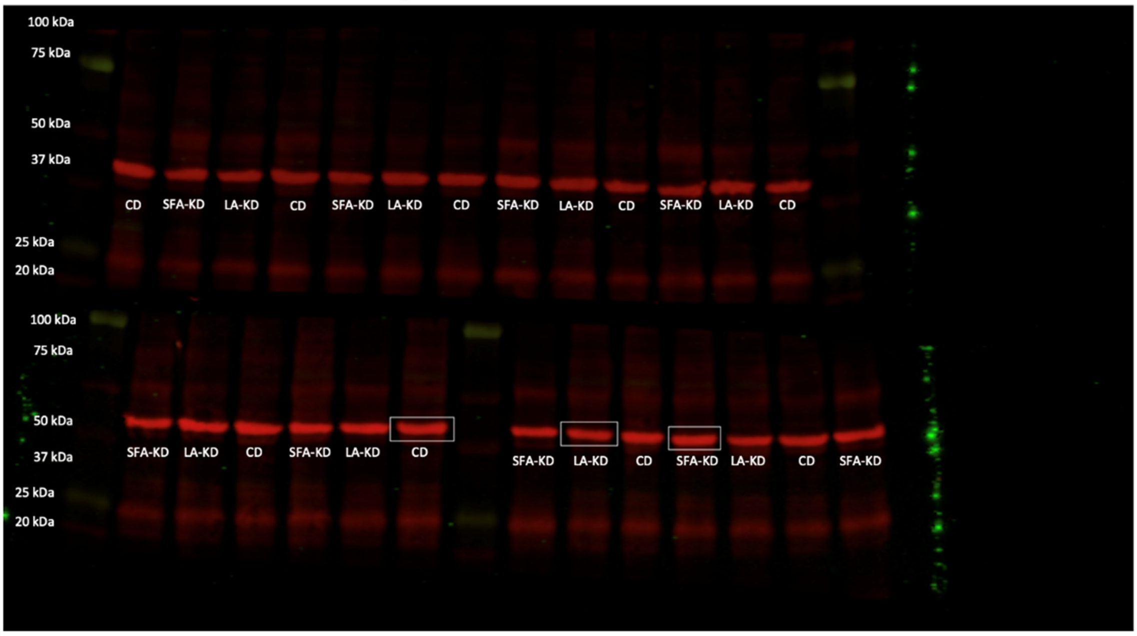

Figure S1. Original and unmodified Western Blot images of jejunal tight junction proteins. A. Claudin-1, B. claudin-2, C. claudin-4, and D. occludin and their loading control (β-actin). The bands presented in the manuscript are circled. CD = control diet, LA-KD = ketogenic diet with linoleic acid, SFA-KD = ketogenic diet with saturated fatty acids.

A

Claudin-1 and occludin

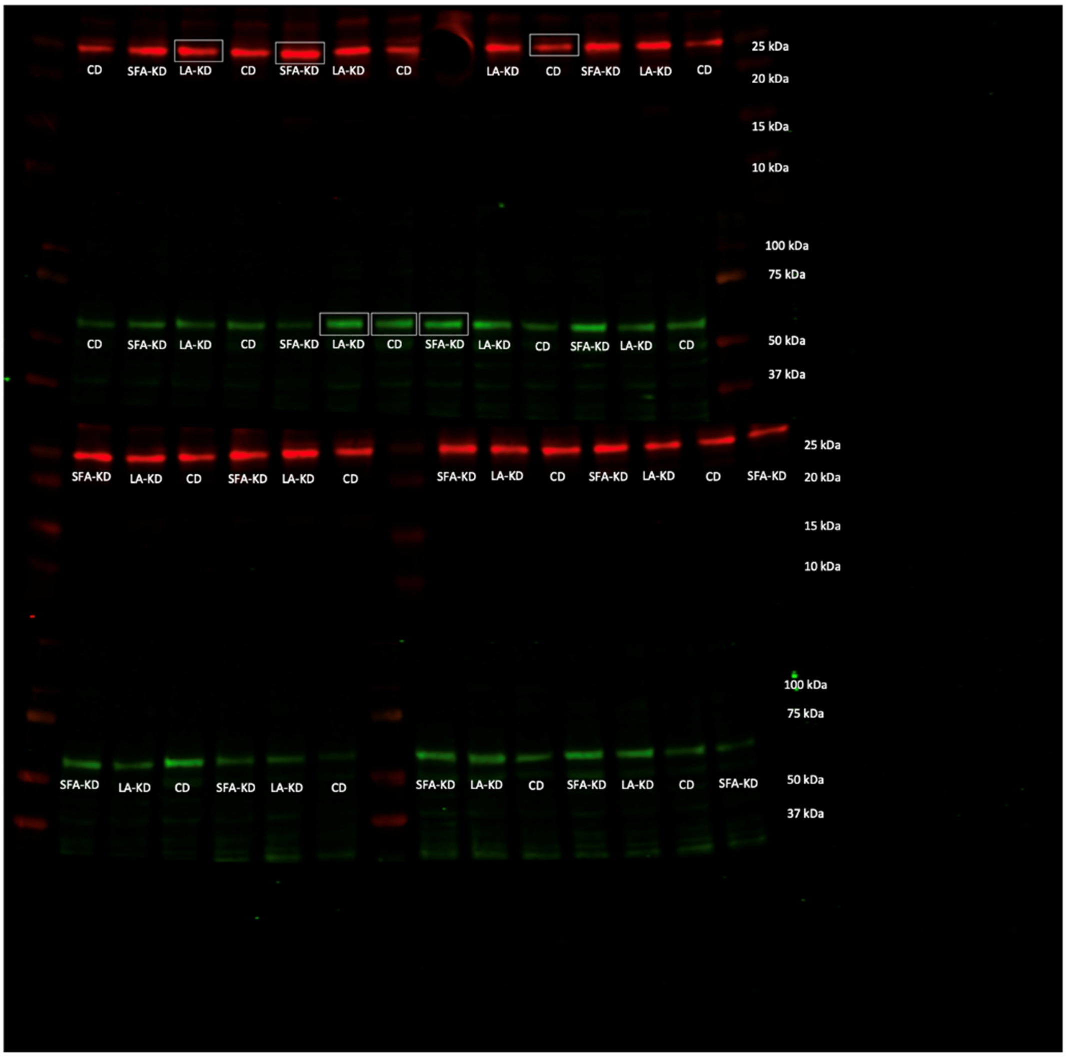

β-actin for claudin-1 and occludin

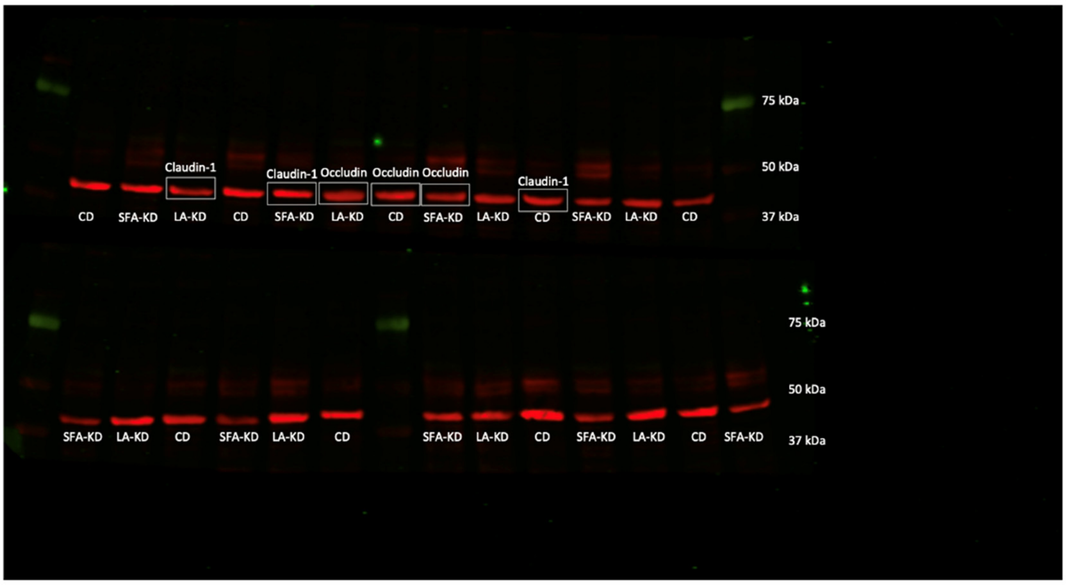

B

Claudin-2

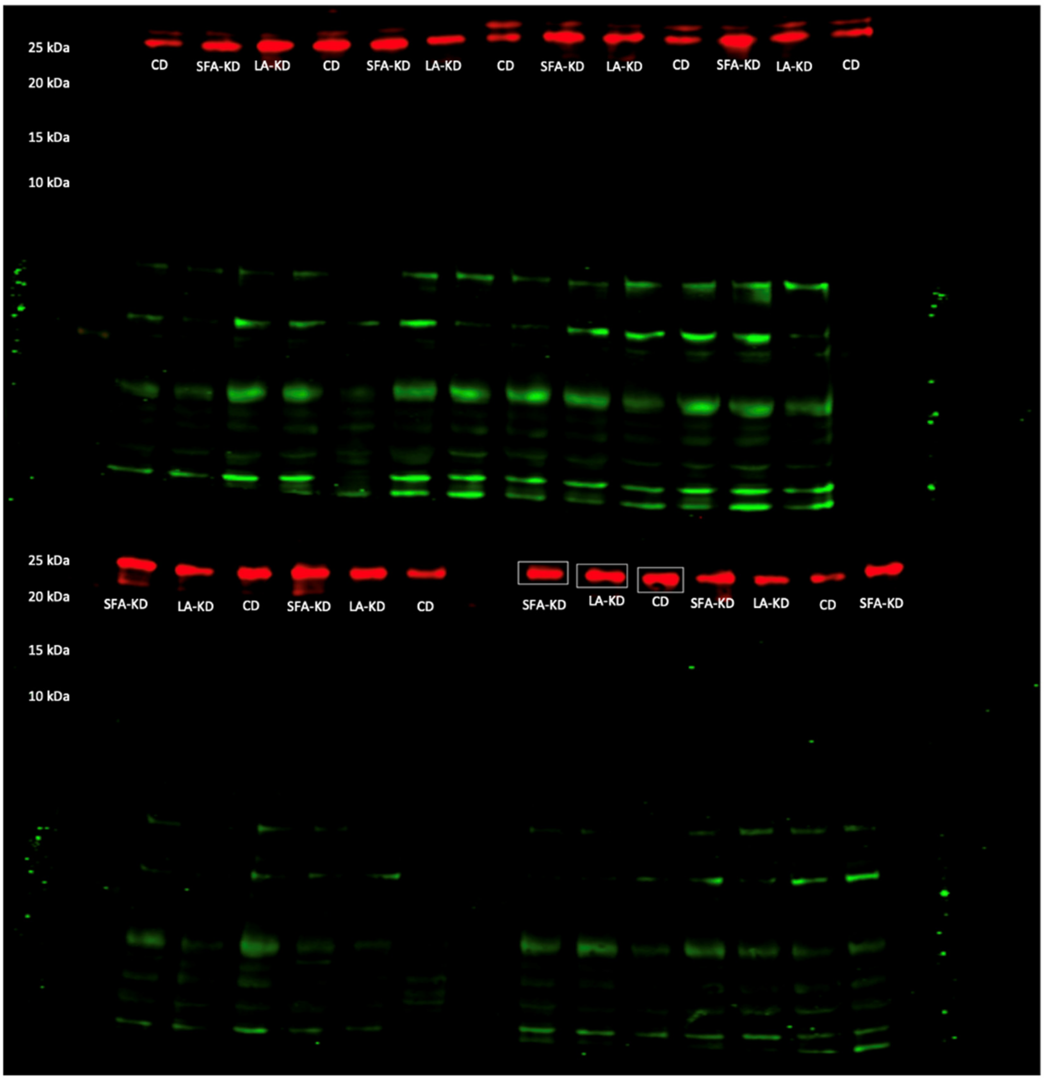

β-actin for claudin-2

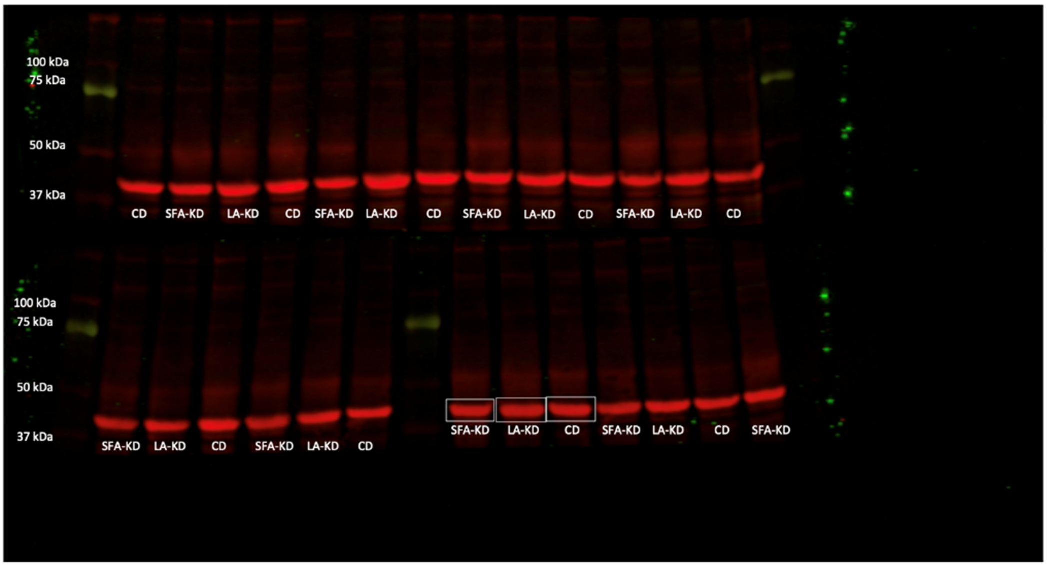

C **Claudin-4**

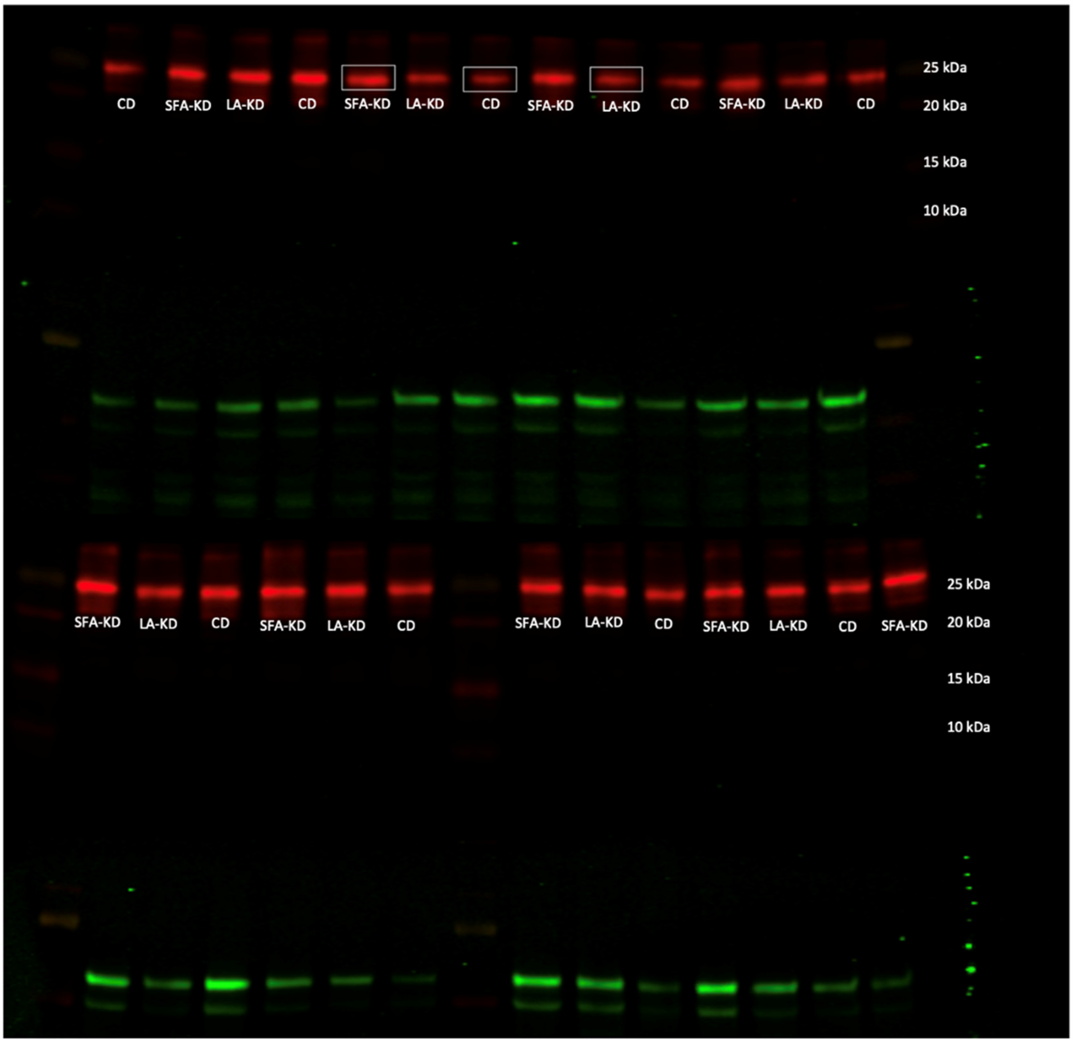

β-actin for claudin-4

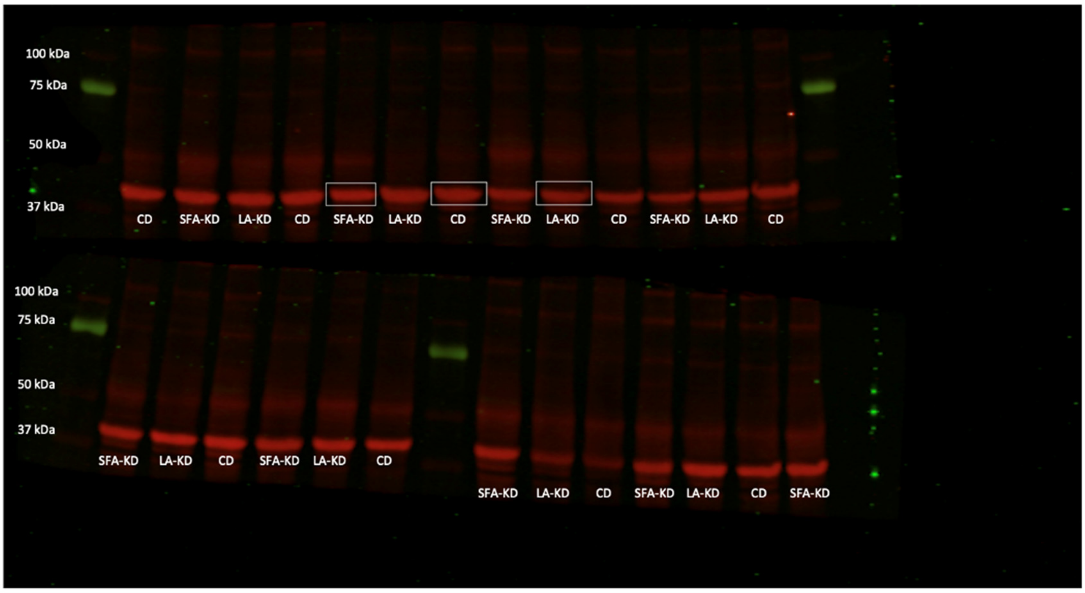

Figure S2. Original and unmodified Western Blot images of colonic tight junction proteins. A. Claudin-1 and occludin, B. claudin-2, and C. claudin-4, and their loading control (β-actin). The bands presented in the manuscript are circled. CD = control diet, LA-KD = ketogenic diet with linoleic acid, SFA-KD = ketogenic diet with saturated fatty acids.
